# Ab interno gel implant in patients with primary open angle glaucoma and pseudoexfoliation glaucoma

**DOI:** 10.1186/s12886-018-0989-6

**Published:** 2018-12-27

**Authors:** Fritz H. Hengerer, Gerd U. Auffarth, Timur M. Yildirim, Ina Conrad-Hengerer

**Affiliations:** 1 Department of Ophthalmology, Ruprecht-Karls-University Heidelberg, 69120 Heidelberg, Germany; 2 International Vision Correction Research Center (IVCRC), University of Heidelberg, 69120 Heidelberg, Germany; 3 University Eye Hospital, Ruprecht-Karls-University, Im Neuenheimer Feld 400, 69120 Heidelberg, Germany

**Keywords:** Pseudoexfoliation glaucoma, Primary open angle glaucoma, Microinvasive glaucoma surgery, XEN45 gel implant

## Abstract

**Purpose:**

To compare efficacy and safety results of an ab interno gel implant in patients with pseudoexfoliation glaucoma (PXG) and primary open angle glaucoma (POAG).

**Methods:**

Retrospective analysis of the medical records of 110 consecutive eyes with open angle glaucoma who had received a XEN45 gel implant between March 2014 and June 2015. Intraocular pressure course, number of glaucoma medications, the need for additional intervention (including needling) and complications were evaluated until 12 months postoperatively.

**Results:**

Data of 67 eyes with POAG and 43 eyes with PXG were analyzed. At 12 months postoperatively, the mean IOP had significantly decreased by 54.0% from preoperatively 31.85 ± 8.5 mmHg to 13.99 ± 2.6 mmHg in the POAG group, (*p* = 0.000; Wilcoxon test), and by 55.2% from 31.63 ± 9.0 mmHg to 13.28 ± 3.1 mmHg in the PXG group (*p* = 0.000; Wilcoxon test). The mean number of anti-glaucoma medications had significantly decreased from 3.25 ± 0.8 at baseline to 0.3 ± 0.7 medications at 12 months postoperatively in POAG eyes (p = 0.000; Wilcoxon test), and from 3.05 ± 1.0 to 0.3 ± 0.6 medications in PXG eyes (p = 0.000; Wilcoxon test). Hypotony (IOP ≤ 6 mmHg) was observed in 2 POAG eyes (3.0%) and in 5 PXG eyes (11.7%) at 1 month but normalized in all eyes at 12 months postoperatively. Severe complications were not observed. No statistically significant differences were found between PXG eyes and POAG eyes.

**Conclusion:**

Our data indicate that the XEN45 gel implant provides significant and comparable reduction in IOP and anti-glaucoma medication during the one-year follow-up period in POAG as well as PXG eyes. This suggests that it may be a noteworthy alternative to traditional filtering procedures in patients with POAG and PXG respectively.

## Introduction

Glaucoma, a progressive optic neuropathy leading to retinal ganglion cell loss and visual field defects, is still one of the leading causes of irreversible blindness [1, 2]. An elevated intraocular pressure (IOP) is a major risk factor for glaucoma development and progression - and lowering IOP is still the only treatment option for glaucoma [3]. Surgical options may be taken into account in patients whose IOP is not sufficiently controllable with two medications and have to be considered if maximally tolerated medical therapy fails [4]. Trabeculectomy is the gold standard filtering surgery with good efficacy; however, it requires a strict postoperative follow-up and has several intra- and postoperative complications [5–7]. In recent years, various micro-invasive glaucoma surgery (MIGS) techniques have been created to provide IOP reduction in a less invasive and safer way [8]. Of these, the XEN45 gel implant (XEN45) (Allergan, California USA) is the only commercially available MIGS device that uses the same outflow pathway as the surgical gold standard trabeculectomy by creating a permanent shunt from the anterior chamber to the subconjunctival space without the need of opening the conjunctiva [8–10]. It is a 6 mm long hydrophilic gelatin tube with an internal diameter of 45 μm that had been designed to maximize long-term outflow while at the same time providing sufficient flow resistance to prevent hypotony [10–13]. Clinical studies have shown that the XEN45 gel implant provides a significant and sustained reduction in IOP and glaucoma medication with a low rate of complications [14–19]. However, most data were collected in patients with primary open angle glaucoma (POAG). In this context, it should be noted that in Europe, the indication of the XEN45 gel implant is limited for patients with POAG, whereas in the USA, the indication also includes the management of refractory glaucoma, including cases where previous surgical treatment has failed, cases of primary open angle glaucoma, and pseudoexfoliative or pigmentary glaucoma with open angles that are unresponsive to maximum tolerated medical therapy [20, 21].

To our knowledge, this is the first study to evaluate and compare the efficacy of the XEN45 gel implant in POAG and pseudoexfoliation glaucoma (PXG) patients with inadequately controlled IOP despite maximized medical therapy or prior glaucoma surgery.

## Methods

This is a retrospective analysis of consecutive patients with PXG and POAG who received a XEN45 gel implant between March 2014 and June 2015. The analysis included only the medical records of patients for whom data on all follow-up visits up to 12 months after surgery were available.

### Patients and assessments

The medical records of 67 eyes with POAG and 43 eyes with PXG were included in the analysis. Patients with an inadequately controlled IOP and optic disc damage despite prior surgical intervention or maximum medication as well as an area of healthy, free and mobile conjunctiva in the target quadrant had received a XEN45 implant. Exclusion criteria were the same as for trabeculectomy: pregnancy, age < 18 years, condition after pars plana vitrectomy, flat anterior chamber and narrow chamber angle. Preoperatively, a complete ophthalmic examination including gonioscopy had been performed. Postoperative evaluations were conducted at day 1, week 1, and months 1, 3, 6, and 12. At each visit, slit-lamp examination, gonioscopy, IOP assessment by Goldmann applanation tonometry (at each assessment, three measurements were performed, the mean value was recorded), as well as evaluation of the posterior pole were carried out. Moreover, the number of medications and adverse events were documented.

### Surgical technique

All implantations were performed by one single surgeon (FHH) following a standardized implantation technique which has been described in detail in a recent publication [14]. The vast majority of XEN implants were performed as a stand-alone procedure. In brief, the XEN45 was implanted under peribulbar anesthesia using an ab interno approach. At first, a volume of 0.1 ml of MMC solution (0.01% mitomycin C, a total dose of 10 μg) was injected subconjunctivally in the nasal superior quadrant to prevent further scarring of the conjunctiva. After the anterior chamber was filled with a medium grade viscoelastic device, the preloaded injector needle was then inserted through a 1.2 mm corneal paracentesis incision opposite the site of desired implantation. The needle was then directed across the anterior chamber and the injector tip was used to penetrate through the chamber angle above trabecular meshwork and the sclera at least 3 mm in length in order to place the implant properly. After careful removal of viscoelastic and hydration of paracenteses the eye was covered with a patch.

Postoperatively, topical antibiotics were given 4 times daily for 10 days in combination with steroids 6 times daily and tapered out over 6 weeks. Anti-glaucoma medication was given until surgery and was completely stopped after the implantation of the gel implant; there was no wash-out phase. At every visit, IOP was assessed and if IOP was elevated, additional anti-glaucoma medication or secondary intervention were given at the discretion of the surgeon. In case of conjunctival scarring and bleb failure due to Tenon’s cyst formation, a needling procedure was performed under microscopic view in the operating room. The needling technique has recently been described in detail [14]. The administration of additional drugs during the needling was considered on a case-by-case basis. Only in cases of pronounced fibrosis (approx. 10% of cases) 10 μg mitomycin C was injected during needling, while in eyes with cystic fibroses no additional drugs were used.

### Statistical analysis

Efficacy outcomes included IOP and the number of anti-glaucoma medications, and their changes as compared to baseline. Additional efficacy outcomes were target IOP of ≤18 mmHg, ≤ 15 mmHg and ≤ 13 mmHg at 12 months. Safety outcomes included hypotony rate (IOP ≤ 6 mmHg), rate of needling, as well as complications. Data were presented as mean and standard deviation, unless otherwise indicated. Baseline IOP was the IOP measured at the preoperative visit on medications. The IOP measured at each visit was then used to calculate the change from baseline. A Kaplan-Meier analysis was performed starting at 1 month postoperatively and using the following criteria for qualified success and complete success: Complete success was defined as an IOP reduction of at least 20% and an IOP value below 18 mmHg without medication. Qualified success was defined as an IOP reduction of at least 20% and an IOP value below 18 mmHg with or without medication. In order to calculate differences between pre- and postoperative values, the parametric T-test was used. Also, the non-parametric Wilcoxon sign rank test was used for determining statistical significance within a group (*p* < 0.05 considered statistically significant). For determining statistical significance between both groups, the non-parametric Mann-Whitney *U* test and the t-Test for independent samples were performed (*p* < 0.05 considered statistically significant). In all other cases chi-square tests were applied (*p* < 0.05 considered statistically significant).

## Results

Overall, medical records of 110 eyes were included in this analysis. Of these, 67 eyes with POAG and 43 eyes with PXG had received a XEN45 gel implant. In the POAG group, the XEN stent was implanted in combination with cataract surgery in eight eyes, while 58 eyes received the XEN stent as standalone procedure. For one patient of the POAG group the information whether the XEN stent was implanted standalone or in combination with cataract surgery was missing. In the PEX group, nine eyes were treated with combined surgery, while 34 eyes received the XEN stent as standalone procedure. The number of eyes was stable at all postoperative visits in both groups. Demographic and baseline characteristics are displayed in Table 1. There was no statistically significant difference regarding demographic and baseline characteristics between the two groups (*p* > 0.05).

**Table 1:** Demographics and baseline characteristics

	POAG (*n* = 67)	PEXG (*n* = 43)
Age (years), mean ± SD (range)	69.6 ± 13.7 (34–91)	74.0 ± 8.3 (51–89)
Gender, n (%)		
Male	26 (38.8)	20 (46.5)
Female	41 (61.2)	23 (53.5)
Operated Eye, n (%)		
OD (right eye)	39 (58.2)	22 (51.2)
OS (left eye)	28 (41.8)	21 (48.8)
Cup to Disc Ratio, mean ± SD (range)	0.82 ± 0.13 (0.4–1.0)	0.80 ± 0.10 (0.6–1.0)
Lens status, n (%)		
Phakic	62 (92.5%)	39 (90.7%)
Pseudophakic	5 (7.5%)	4 (9.3%)
Prior glaucoma intervention, n (%)		
none	22 (32.8%)	14 (32.6%)
Trabeculectomy	13 (19.4%)	6 (14.0%)
Laser	4 (6.0%)	0 (0.0%)
CPC	13 (19.4%)	6 (14.0%)
Microstent^a^	10 (14.9%)	13 (30.2%)
Phacoemulsification	5 (7.5%)	4 (9.3%)
IOP (mmHg), mean ± SD (range)	31.85 ± 8.5 (20–65)	31.63 ± 9.0 (20–59)
Number of medication, mean ± SD (range)	3.25 ± 0.8 (2–5)	3.05 ± 1.0 (0–5)

*POAG* primary open angle glaucoma, *PEXG* pseudoexfoliation glaucoma, *CPC* Cryophotocoagulation, *IOP* intraocular pressure, *SD* standard deviation

^a^other than XEN gel implant

**Fig. 1:**
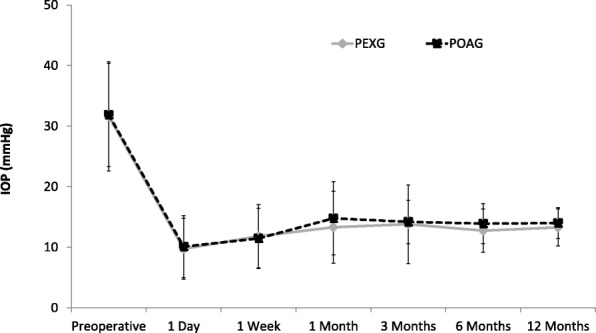
Mean IOP at each study visit in PXG eyes (*n* = 43) und POAG eyes (*n* = 67) respectively. Error bars indicate SD for the mean. Within each group, the mean IOP was significantly reduced from baseline at any visit during the follow-up period (*p* = 0.000; Wilcoxon test). Between groups, no significant differences were observed (*p* > 0.085 at each visit; t-Test)

**Fig. 2:**
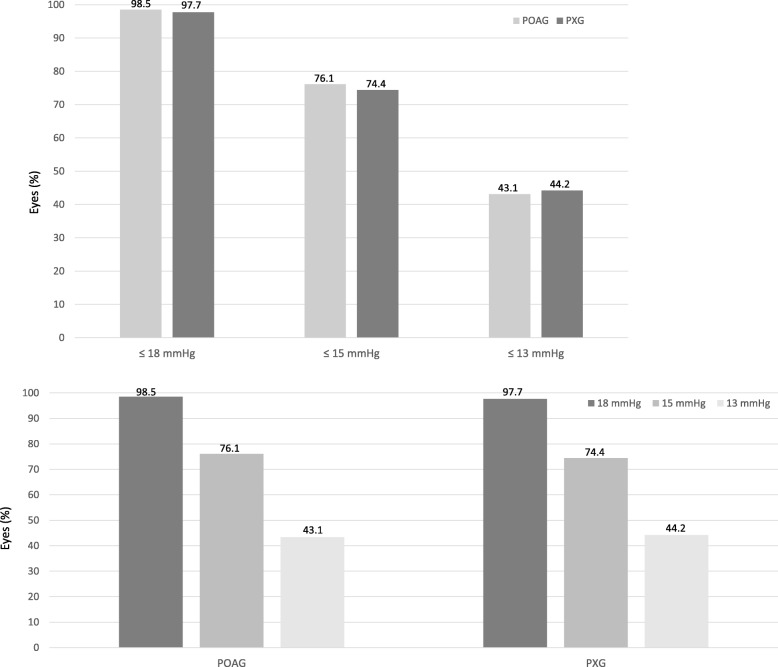
Proportion of eyes reaching a certain target pressure at 12 months postoperatively. Comparison of eyes with POAG or PXG

Both groups showed a significant reduction in IOP, which started on the first day after surgery and continued for the entire follow-up period of one year. At 12 months postoperatively, the mean IOP had significantly decreased by 54.0% from preoperatively 31.85 ± 8.5 mmHg to 13.99 ± 2.6 mmHg in the POAG group, (*p* = 0.000; Wilcoxon test), and by 55.2% from 31.63 ± 9.0 mmHg to 13.28 ± 3.1 mmHg in the PXG group respectively (*p* = 0.000; Wilcoxon test). No significant differences between the two groups were detected at any time during follow-up observation (*p* > 0.085 at each postoperative visit; t-Test) (Fig. 1). The proportion of eyes per group that achieved target pressure values of 18 mmHg, 15 mmHg and 13 mmHg one year postoperatively is shown in Fig. 2.

**Fig. 3:**
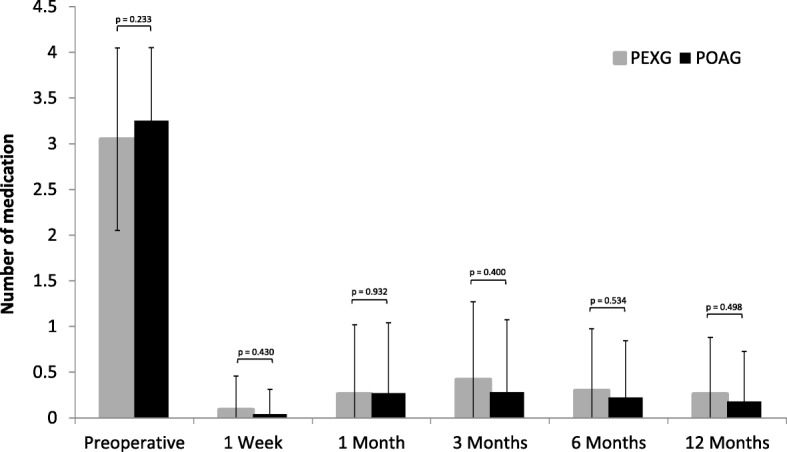
Mean number of medication at each study visit in PXG eyes (*n* = 43) und POAG eyes (*n* = 67) respectively. Error bars indicate SD for the mean. Within each group, the mean number of medication was significantly reduced from baseline at any visit during the follow-up period (*p* = 0.000; Wilcoxon test). Between groups, no significant differences were observed (*p* > 0,233 at each visit)

The anti-glaucoma medication was reduced in both groups in the median from preoperative 3 medications to 0 medications in the entire postoperative course. The mean number of anti-glaucoma medications had significantly decreased from 3.25 ± 0.8 at baseline to 0.3 ± 0.7 medications at 12 months postoperatively in POAG eyes (*p* = 0.000; Wilcoxon test), and from 3.05 ± 1.0 to 0.3 ± 0.6 medications in PXG eyes (*p* = 0.000; Wilcoxon test). No significant differences between the groups were observed at any time during follow-up (*p* > 0,4 at each postoperative visit) (Fig. 3). At 12 months postoperatively, 88.1% of POAG eyes and 83.1% of PXG eyes were completely off drops.

**Fig. 4:**
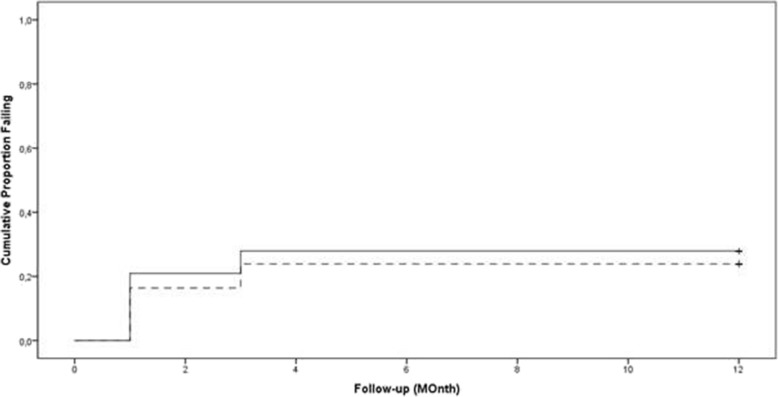
Kaplan-Meier plots of the cumulative probability of failure using the qualified success criteria. (≥20% IOP reduction and an IOP of less than 18 mmHg with or without medication and/or without any secondary intervention). The curve indicates that 76.1 and 72.1% of eyes with POAG (dotted line) and PXG (solid line) respectively achieved qualified success until 12 months postoperatively. The difference was statistically not significant (*p* = 0.626; LogRank)

**Fig. 5:**
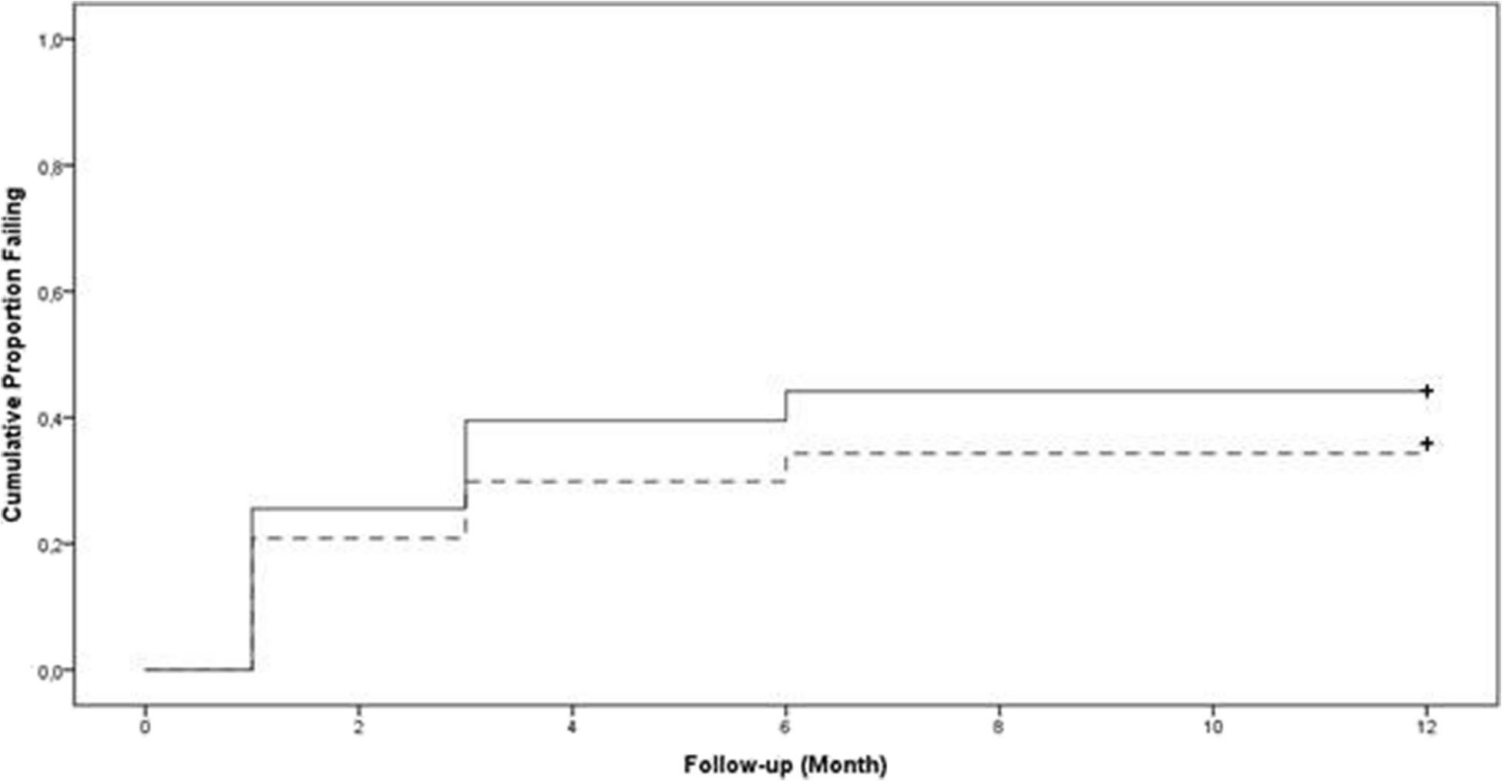
Kaplan-Meier plots of the cumulative probability of failure using the complete success criteria. (≥20% IOP reduction and an IOP of less than 18 mmHg with or without medication and/or without any secondary intervention). The curve indicates that 64.2 and 55.8% of eyes with POAG (dotted line) and PXG (solid line) respectively achieved complete success until 12 months postoperatively. The difference was statistically not significant (*p* = 0.374; LogRank)

One year after XEN45 implantation, 76.1% of eyes with POAG and 72.1% of those with PXG had achieved qualified success (IOP reduction of at least 20% and an IOP value below 18 mmHg with or without medication). Complete success (IOP reduction of at least 20% and an IOP value below 18 mmHg without medication) was experienced by 64.2% of POAG eyes and by 55.8% of PXG eyes. The results of the Kaplan-Meier analysis using qualified success criteria and complete success criteria are shown in Figs. 4 and 5.

Overall, we had 28 cases of failure (i.e. IOP > 18 mmHg and/or IOP reduction < 20%; with or without medication) after 12 months due to reduced filtration by late fibrosis of the bleb area. Of these, there were 16 failures in the POAG group and 12 failures in the PXG group.

In the early postoperative phase, between week 1 and 3 months, 29.9% eyes in the POAG group required needling to enhance the outflow, while this was the case in 34.9% of PXG eyes respectively. The difference between both groups was not statistically significant (*p* = 0.058; t-Test). Twelve of 110 eyes received a single MMC injection between the first and third month (7 POAG eyes and 5 PXG eyes). Hypotony (IOP ≤ 6 mmHg) was observed in 2 POAG eyes (3.0%) and 5 PXG eyes (11.7%) at 1 month (*p* = 0.108; Fisher’s exact test). Neither of the two groups had any cases of wound leakage, corneal alterations, device exposure or migration, choroidal effusion or hemorrhage, choroidal detachment, or endophthalmitis during the follow-up.

## Discussion

The purpose of our retrospective data analysis was to evaluate and compare the efficacy and safety results of the XEN45 gel implant in patients with PXG and POAG. Within the last years, various MIGS devices have been developed with the aim to provide less invasive methods of decreasing IOP in glaucoma patients than traditional surgery and reducing the patients’ dependency on topical medications [8]. Several clinical studies have shown that the XEN45 gel implant provides a significant and enduring reduction of IOP and anti-glaucoma medication [14–19]. Since these studies have focused on POAG patients, very little data is available on PXG, the most common secondary glaucoma [22]. However, because of the aggressive course of the disease and their poor response to medical therapy [23], PXG patients in particular could probably benefit from an early and less invasive intervention.

Our results show that in both patient groups a strong and comparable IOP decrease as well as reduction of anti-glaucoma medication can be achieved with the XEN45 gel implant. In POAG eyes as well as in PXG eyes, we observed a significant reduction in the mean IOP by more than 50% to a mean IOP below 15 mmHg at each postoperative visit. In both groups, the IOP lowering effect started on the first postoperative day and continued throughout the follow-up period. Additionally, in both patient groups the mean number of anti-glaucoma medications was significantly reduced after XEN45 implantation and more than 80% of eyes were completely off drops. This substantial reduction of anti-glaucoma medication achieved in both patient groups may contribute to a better quality of life and can increase patients’ satisfaction with their therapy [24]. At the same time, the XEN45 had a reliable safety profile with no severe complications. We did not observe any significant differences regarding efficacy and safety between the two groups. However, even if the differences were not statistically significant, hypotony of less than 6 mmHg was more frequent in PXG eyes one month postoperatively. This may be due to the fact that some pseudoexfoliative material has been removed by rinsing the anterior chamber during implantation of the XEN45, thus improving fluid drainage. Nevertheless, all cases of hypotony were resolved after six months and no choroidal effusion was observed.

A total of 17 eyes in our study received the XEN implant in combination with cataract surgery (8 POAG eyes, 9 PXG eyes). With regard to IOP and number of medications at baseline and after 12 months we could not find any statistical differences between the different subgroups, however, since the number of combined cases is very low (< 10 in the respective subgroups), these results should be viewed with the utmost caution and should not be overinterpreted.

Our results are well in line with data from other clinical studies evaluating the efficacy and safety of the XEN45 gel implant in patients with POAG [14–19]. Moreover, our results, collected in 43 eyes with PXG, a very similar to the data from Ilveskoski and Tuuminen who report clinical results of XEN45 gel implant in 10 patients with PXG. In this group of patients, the mean IOP was reduced by 47.4% from 33 mmHg preoperatively to 10.2 mmHg at six months postoperatively and mean medication had decreased from 2.4 to 0.9 medications [25].

As the XEN45 gel implant uses the same outflow path as the surgical gold standard trabeculectomy, our study results are not comparable to those of other MIGS devices which target the natural outflow pathways of aqueous humor. Overall, our results indicate a strong IOP-lowering effect after XEN45 implantation which can be achieved in POAG patients as well as in PXG patients and is almost comparable to that of conventional filtrating glaucoma surgery [6], although however, in the absence of a prospective, randomized trial, it is difficult to draw any major conclusions versus trabeculectomy. At the same time, typical safety risks of traditional incisional glaucoma surgery, such as hypotony-related complications, scarring, foreign body reaction, cataract formation, and surgically induced astigmatism are almost negligible in this ab-interno procedure. Therefore, given the marked and enduring IOP-lowering effect of the XEN45 and its reliable safety profile, this micro-invasive procedure might be considered at an earlier stage of glaucoma disease than conventional filtrating glaucoma surgery. Especially in PXG patients, who often respond only weakly to medical therapy and exhibit rapid disease progression [23], this approach may help to achieve a low target IOP with reduced medication at an earlier stage. Moreover, this strategy would preserve the conjunctiva if further glaucoma filtrating surgery interventions might be required to control IOP more efficiently.

Our study has limitations that should be addressed. First, it was a retrospective analysis of single center data, however, this setting reflects the everyday clinical routine and thus provides important insights into the efficiency and safety of the XEN45 gel implant in everyday clinical practice. Secondly our results are only out to 12 months which is a relatively short time in a lifetime chronic disease. Thirdly no consistent follow up of perimetry data was available, as preoperative perimetry assessments had been performed in the respective private practices, before the patients were referred to our clinic. Nevertheless, all patients appeared at our department with clinical signs of progression or uncontrolled IOP values, respectively.

However, despite its limitations, this study contributes to increasing evidence showing the safety and efficacy of the XEN45 gel implant. This procedure provided significant and comparable reduction in IOP and anti-glaucoma medication during the entire follow-up period in POAG and PXG eyes, suggesting that it might be a noteworthy alternative to traditional filtering procedures in patients with POAG and PXG respectively**.** To confirm these one-year results, further prospective, randomized studies with longer follow-ups are required to evaluate the efficacy and safety of this procedure in PXG patients.

## Abbreviations

CPC: Cryophotocoagulation
IOP: Intraocular pressure
MIGS: Minimally/ Micro-invasive glaucoma surgery
MMC: Mitomycin C
POAG: Primary open angle glaucoma
PXG: Pseudoexfoliation glaucoma
